# Green Processing of Black Raspberry Pomace: Application of Sonotrode-Based Extraction Technique and Particles from Gas-Saturated Solutions (PGSS) Technology

**DOI:** 10.3390/foods12203867

**Published:** 2023-10-22

**Authors:** Nataša Nastić, Zorana Mutavski, Jelena Živković, Rita Ambrus, Naiara Fernández, Nebojša Menković, Senka Vidović

**Affiliations:** 1 Department of Pharmaceutical Engineering, Faculty of Technology Novi Sad, University of Novi Sad, Bulevar Cara Lazara 1, 21000 Novi Sad, Serbia; natasa.nastic@uns.ac.rs (N.N.); zmutavski@mocbilja.rs (Z.M.); 2 Institute for Medicinal Plants Research “Dr Josif Pančić”, Tadeuša Košćuška 1, 11000 Belgrade, Serbia; jzivkovic@mocbilja.rs (J.Ž.); nmenkovic@mocbilja.rs (N.M.); 3 iBET, Instituto de Biologia Experimental e Tecnológica, Apartado 12, 2781-901 Oeiras, Portugal; ambrus.rita@szte.hu; 4 Institute of Pharmaceutical Technology and Regulatory Affairs, Faculty of Pharmacy, University of Szeged, H-6720 Szeged, Hungary; naiara.fernandez@ibet.pt

**Keywords:** black raspberry pomace, UAE, anthocyanins, PGSS, morphology

## Abstract

The aim of this study was to develop, for the first time, anthocyanin-enriched fractions from black raspberry pomace (BRP) using ultrasound-assisted extraction (UAE) via sonotrode and the Particles from Gas-Saturated Solutions (PGSS) process. UAEs with different amplitudes and sonication times were evaluated and showed relevant effects on the yields of target analytes. The raspberry pomace extracts were formulated in a powder form by PGSS using glyceryl monostearate as a carrier at different extract-to-carrier ratios of 1:11, 1:5, and 1:3. The effects of all variables were evaluated in terms of extraction yield, total phenolic content, and encapsulation yield. UAE was strongly affected by amplitude, and the highest amplitude (100%) provided the best results for extraction yield and total phenolics. HPLC of UAE extracts and powders was utilized for quantification of polyphenol compounds, showing cyanidin-3-rutinoside as a main compound, followed by cyanidin-3-glucoside, rutin, ellagic acid, and gallic acid. These results show that these time-efficient and high-performance techniques enable the production of natural fractions from industrial BRP with acceptable characteristics to be used for the development of nutraceuticals and different food formulations.

## 1. Introduction

Current trends in food and nutraceutical research are geared toward technologies that are specific, highly efficient, and cost-effective. Recently, studies have been focusing on novel methodologies that involve a sequence of unit operations. This approach begins with utilizing agro-industrial residues to extract specific bioactive compounds through environmentally friendly extraction methods. Subsequently, these extracted compounds are processed into particles, creating ingredients suitable for use in functional foods and nutraceutical applications [1,2].

Globally, the assessment of agro-industrial residues shows that the industrial processing of fruits generates around 40% of total waste and by-products in the form of pomace, including skins, seeds, and, occasionally, stalks, or damaged fruits [3]. The use of material such as pomace is related to the circular and greener economy, as suggested by goal 12 of the 17 Sustainable Development Goals set by the United Nations. Raspberry pomace is a nutrient-dense ingredient with a wide range of functions. The literature shows that the raspberry pomace consists mainly of seeds (80%) and pulp as a source of essential fatty acids, polyphenols, anthocyanins, ellagic acid, ellagitannins, and tocopherols [4,5]. Anthocyanins from berry pomace are recognized for their significant antioxidant, antiproliferative, proapoptotic, and antibacterial properties [6,7].

Global raspberry production has increased from 797,048 tons in 2017 to 886,538 tons in 2021 [8], and during processing, more than one-third of berries remain discarded. The application of methodologies able to analyze raspberry pomace compounds readily is paramount in the current agro-industrial waste scenario to allow the exploration of their potential. In this context, the application of green-based processes—ultrasound-assisted extraction (UAE) with supercritical carbon dioxide-assisted encapsulation—is a potential tool to design and produce natural ingredients from fruit by-product fractions with completely new specifications. This approach follows the principles of Green Chemistry and the Circular and Bioeconomy concept by using discarded biomass to create new or more advanced (semi)products [9,10,11]. UAE, regarded as a non-conventional technology, stands out as a promising alternative for extracting bioactive compounds from plants. UAE is a well-established green bio-refining technique, primarily due to its cost-effectiveness; its superior performance with reduced solvent, time, and energy requirements; and its suitability for heat-sensitive compounds [12,13,14]. However, after extraction, additional processing methods could potentially improve the physicochemical stability and extend the shelf life of the extract. Conventional extract processing often involves heat treatments with potential negative impacts on the stability of anthocyanins, as they are very sensitive to degradation caused by exposure to higher temperatures, light, oxygen, and enzymes, and they are strongly affected by pH change. The particles from gas-saturated solutions (PGSS) process is one of the solutions that fits into the green technology’s framework. The PGSS process can be used as an alternative to conventional spray-drying, avoiding high processing temperatures (an important parameter in the case of anthocyanin/phenol drying), long processing times, significant mechanical stress, and issues related to residual organic solvents. The sensitivity of supercritical carbon dioxide to minor temperature and pressure variations within its highly compressible region offers the potential to finely tune both particle size and structure across a broad spectrum with only minor adjustments to process conditions.

To the best of our knowledge, there is no information in the literature about the abovementioned using green-based processes for black raspberry pomace (BRP) valorization. Therefore, from the perspective of sustainable development, our aim was to produce an anthocyanin-rich extract from BRP and to formulate it in an acceptable form to provide protection to the anthocyanins and prolong their stability. For extraction and formulation, UAE via sonotrode and the PGSS technique were used. UAEs with different amplitudes and sonication times were evaluated in order to maximize the extraction of bioactive compounds from BRP, while the effect of microencapsulation conditions (carrier material and the ratio of BRP extract and coating material) on the physical and chemical properties of BRP powder were also investigated. The identification and quantification of the phenolic compounds of the extracts in both encapsulated and unencapsulated forms were carried out.

## 2. Materials and Methods

### 2.1. Plant Material and Chemicals

Fresh raspberry pomace was provided by the agricultural holding Grujo Jovanović (Godečevo, Serbia). The raw material was subjected to drying in a vacuum dryer and subsequently ground using a blender. The average particle size was found to be 0.76 ± 0.06 mm, with a moisture content of 6.40 ± 0.13%. The dried material was stored in sealed packages away from light and used in all further analyses.

Folin–Ciocalteu reagent, gallic acid, acarbose, amylase (from the porcine pancreas), and formic acid were purchased from Sigma-Aldrich (Steinheim, Germany). Ethanol (EtOH) was purchased from Centrohem (Šabac, Serbia). Acetonitrile (Merck, Darmstadt, Germany) was of HPLC grade, and bi-distilled water was prepared using a Milli-Q purification system (Millipore, Molsheim, France). Imwitor^®^ 600 was kindly supplied by Sasol (Witten, Germany). Glyceryl monostearate (GMS) was purchased from Gattefossé SAS (Saint-Priest, France). All the standard phenolic compounds (grade purity > 96%) were purchased from Extrasynthese (Cedex, Genay, France). All other chemicals were of analytical grade and were used without further purification.

### 2.2. Conventional Solid–Liquid Extraction

The conventional solid–liquid extraction (SLE) process involved the maceration of BRP with aqueous EtOH mixtures (30, 50, and 70%) [7]. All SLE experiments were conducted at a temperature of 25 °C for a duration of 24 h using a temperature-controlled shaker (KS 4000i, IKA, Staufen, Germany) set at 150 rpm. Dried BRP (10 g) was mixed with solvent at a solvent-to-sample ratio of 1:10 (*m*/*v*). Following extraction, the resulting crude extracts were promptly filtered through filter paper using a vacuum apparatus (V-700, Büchi, Flawil, Switzerland), collected into vials, and stored at 4 °C until further analysis.

### 2.3. Ultrasound-Assisted Extraction via Sonotrode

In order to investigate and characterize the impacts of ultrasound treatment, a set of parameter variations was designed. Ultrasonic treatment of raspberry pomace using 10 g of dried BRP and 30% EtOH as a solvent at a solvent-to-sample ratio of 1:10 (*m*/*v*) was carried out directly in a glass beaker using an ultrasonic homogenizer (UP 400St, 400 W, 24 kHz, Hielscher GmbH, Stuttgart, Germany). A titanium ultrasonic probe d14 (Hielscher GmbH, Stuttgart, Germany) was used, functioning in continuous mode. Extraction times of 2, 4, 6, 8, and 10 min and amplitudes of 20, 60, and 100% were applied. Changes in temperature, power, and energy consumption depending on time were monitored during the extraction. After extraction, the extracts were filtered under a vacuum, collected in vials, and stored at 4 °C until analysis.

### 2.4. Particles from Gas-Saturated Solutions Process

Microparticles derived from the BRP extract were generated using the PGSS^®^ technique. The dispersion was formulated in a temperature-controlled, high-pressure stirred vessel (PGSS^®^ Separex Supercritical and High-Pressure Technology, Champigneulles, France) at 65 °C by adding the 1 mL of emulsifier (Imwitor^®^ 600) to 3 g of a mixture of BRP extract and GMS (carrier). The mass ratios of the BRP extract to carrier tested were 1:11, 1:5, and 1:3. Carbon dioxide was delivered into a 50 cm^3^ electrically thermostated, high-pressure stirred vessel using a high-pressure piston pump (29723-71, Haskel International Inc., Burbank, CA, USA) until the desired operating pressure (100, 150, or 200 bar) was achieved. After a period of equilibrium at 150 rpm, an automated depressurization valve was employed to release the pressure, and the mixture was atomized through a two-fluid nozzle with a diameter of 710 μm and external mixing (Spraying Systems Co., Air atomization 1/4J-SS, Separex, Champigneulles, France). This atomized material was then combined with compressed air at a pressure of 7 bar to enhance the drying process within a cyclone. Finally, the particles were collected in an 18 L vessel at atmospheric pressure. The encapsulation yield (EnY) was determined by dividing the amount of the encapsulated powder obtained at the end of the process by the initial sample amount loaded into the vessel.

### 2.5. Total Phenolic Content

Total phenolic content (TPC) was quantified through a colorimetric assay using the Folin–Ciocalteu method. This method relies on the oxidation and reduction reactions of phenolic compounds [15]. The absorbance was measured at 750 nm, and gallic acid was used for the calibration curve (y = 89.04762x + 0.01358, *R*^2^ = 0.9992). The results are expressed as mg GAE/g dry weight (DW). The analyses were performed in triplicate.

### 2.6. HPLC Analysis

The analysis of individual anthocyanin compounds and rutin was performed using an Agilent 1200 RR system (Agilent, Waldbronn, Germany) equipped with a diode array detector. A reversed-phase Lichrospher RP-18 (Agilent) column (250 mm × 4 mm, 5 μm) was used with the column temperature maintained at 25 °C. The mobile phase consisted of solvent A (10%, *v*/*v* solution of formic acid in water) and solvent B (acetonitrile) using the following gradient elution profile: 1% B 0–0.5 min; 1–7% B 0.5–1 min; 7% B 1–4 min; 7–10% B 4–7.5 min; 10–14% B 7.5–11.5 min; 14–25% B 11.5–15.5 min; 25–40% B 15.5–18.5 min; 40–75% B 18.5–22 min; 75% B 22–25 min. The injection volume was 10 μL, the flow rate was 1 mL/min, and detection was carried out at wavelengths of 290, 350, and 520 nm. The compound contents were determined using calibration curves, and the results are expressed as milligrams per gram of dry weight (mg/g DW). The analyses of gallic and ellagic acids were conducted using an Agilent 1200 RR system (Agilent, Waldbronn, Germany) equipped with a diode array detector. A reversed-phase Zorbax SB-C18 (Agilent) column (150 mm × 4.6 mm, 5 μm) was used, and the column temperature was maintained at 25 °C. The mobile phase consisted of solvent A (1%, *v*/*v* solution of orthophosphoric acid in water) and solvent B (acetonitrile) using the following gradient elution profile: 0–5 min, 98–90% A; 5–15 min, 90% A; 15–20 min, 90–85% A; 20–25 min, 85–70% A; 25–30 min, 70–40% A; 30–34 min, 40–0% A, with a post-time of 2 min and a flow rate of 1 mL/min. The injection volume was 3 μL, and detection was carried out at wavelengths of 260 and 320 nm. Compound content was determined using calibration curves. The results are expressed as milligrams per gram of dry weight (mg/g DW). The calibration curve correlation coefficients ranged from 0.9989 to 0.9999, and the target compounds showed good linearity within the range of 100–650 µg/mL for anthocyanin compounds and from 50 to 500 µg/mL for other investigated polyphenols.

### 2.7. Powder Characterization

#### 2.7.1. Particle Size

The average particle size of the microparticles was assessed using the Leica Image Processing and Analysis System device (Leica Q500MC; Leica Microsystems, Wetzlar, Germany). The analysis involves the measurement of parameters for 300 particles, including their length, width, area, district/convex perimeter, bulk and tapped density, Carr index, and Hausner ratio.

To determine the densities, the samples were placed in tared 5 mL graduated cylinders, and the bulk density (ρ_bulk_) values were calculated as mass/volume ratios. Tapped density (ρ_tap_) values were obtained using the STAV 2003 Stampf volumeter (Engelsmann A.G., Ludwigshafen, Germany) as the tapping equipment, and the results were calculated as mass/tapped volume ratios. The method for measuring the bulk and tapped density of powders is outlined in the European Pharmacopoeia, chapter 2.9.34. The compressibility index (Carr index) and the Hausner ratio are parameters that assess a product’s ability to settle and provide insights into the relative significance of interparticle interactions [16]. They were determined by Equations (1) and (2):

Carr index = 100 (ρ_tap_ − ρ_bulk_)/ρ_tap_ (1)

Hausner ratio = ρ_tap_/ρ_bulk_ (2)

#### 2.7.2. Scanning Electron Microscope (SEM)

The morphology of the resulting powder particles was investigated using SEM (Hitachi S4700, Hitachi Scientific Ltd., Tokyo, Japan) at 10 kV. The distribution of the LAM particle diameter was determined through the analysis of SEM images.

### 2.8. Statistical Analysis

All analyses were conducted in triplicate, and the results are presented as means ± standard deviation (SD). To determine significant differences between the results, a one-way ANOVA statistical analysis was performed, followed by the Tukey test. Differences were regarded as significant when *p* < 0.05.

## 3. Results and Discussion

### 3.1. Characterization of BRP Extracts

#### 3.1.1. Extraction Yield and Total Phenolic Content of BRP Extracts

SLE was performed as a reference technique for the selection of the optimal solvent for the extraction of bioactive polyphenolic compounds from BRP. The extraction yield (EY) was expressed as the mass of dry extract (g) per g of dry plant material, i.e., the percentage (%). The results of SLE demonstrated that the EY values were in the range of 15.37–16.46%, while the TPC ranged from 31.50–37.97 mg GAE/g DW (Table 1). The highest EY (16.46%) and TPC (37.97 mg GAE/g DW) were achieved under the same conditions (30% EtOH). EtOH was demonstrated to be less efficient in terms of TPC when used at higher concentrations, probably due to the solvation provided by the water in the mixture [7]. Since there was no significant difference between the measured EYs using different concentrations of EtOH and the TPC was the highest in the extract obtained with the lowest EtOH concentration, 30% EtOH was selected as the optimal solvent for UAE.

According to Živković et al. [17], UAE is an extraction technique that reduces the time needed to obtain high-quality extracts, and it is a trouble-free operating procedure with environmentally friendly features, among others. Since the performance of applied UAE is influenced by various processing parameters, the amplitude and extraction time were specifically examined as the main factors expected to impact the recovery levels. The results in Table 1 reveal that all the variables studied significantly influenced the concentration of total phenols in the extracts since their *p*-values were below 0.05. The highest EY (16.09%) and TPC (44.74 mg GAE/g DW) in UAE were achieved in a significantly shorter extraction time compared with the reference (6/4 min vs. 24 h), accomplishing a rapid environmentally friendly procedure with low energy consumption. An increase in the extraction times, between 2 and 10 min, exhibited higher EYs and TPCs in BRP, aligning well with the results reported by Mutavski et al. [7], who described the same trend in black elderberry pomace extracts obtained by UAE. Similarly, both EYs and TPCs increased with higher ultrasound intensity. This trend could be attributed to enhanced diffusivity resulting from cell disruption and fragmentation induced by the intensified implosion of cavitation bubbles [18]. An additional factor contributing to the increase in EY is the mechanical vibration of the ultrasonic probe, which results in an expanded contact area between the solid material and the solvent. This, in turn, enhances the solvent’s penetration into the matrix [19]. The extraction process using the lowest amplitude (20%) should be considered the main factor contributing to the lowest EYs and TPCs of BRP extracts. After 4 min and at 100% amplitude, a slight decrease in the TPC became noticeable. Therefore, extending the extraction time further might lead to a decrease in the extraction of the target compound. Being exposed to ultrasound at higher amplitudes for long periods causes structural damage to more heat-labile phenolics and/or promotes other chemical changes, which was confirmed during the extraction of polyphenolic compounds from red raspberries [20] and purple corn pericarp [21].

An increase in amplitude also results in a higher process temperature unless it is regulated by a cooling system. Considering the nature and sensitivity of phenolic compounds, maintaining the process temperature within a specific range can be advantageous, as it helps prevent the heat-induced conversion of phenolics. By monitoring the temperature, after 6 min and at 100% amplitude, UAE was stopped due to phenolic compounds’ susceptibility to higher temperatures (over 80 °C) and their possible degradation, which was confirmed by a slight decrease in the TPC. The increase in temperature when using ultrasound was slower at lower amplitudes; operating temperatures using 20% amplitude were ca. 10–20 °C lower than those at higher amplitudes (Table S1).

The results observed in this study were comparable to those reported by Kryževičiūtė et al. [22] for the EY and TPC, ranging from 21.6–25.1% and 26.31–36.18 mg GAE/g DW. Pressurized liquid extraction using methanol was optimized to obtain the hydrophilic fraction from ground raspberry pomace. On the other hand, studies conducted by Krivokapić et al. [23] using an ultrasonic cleaner for 120 min at 50 °C and 50 kHz showed a significantly lower TPC (27.79 mg GAE/L of extract) in comparison with the results obtained in this study. More than eightfold lower concentrations of polyphenols were reported in a study conducted by Saad et al. [24] using an aqueous enzyme-assisted extraction to simultaneously recover lipophilic compounds and polyphenols from raspberry pomace.

For a more accurate comparison, a systematic study was necessary because the yield and second metabolite composition of the extract can vary depending on the fruit sample itself, mass transfer, and extraction technique [17,25]. Park et al. [25] demonstrated that two representative varieties of black raspberry exhibited different metabolic profiles despite their close proximity in cultivation locations. Furthermore, the metabolome of black raspberry fruit from the same variety can differ depending on environmental and cultivation conditions, including variations in temperature, rainfall, sunlight exposure, soil composition, and the biological environment of the cultivation sites [25,26].

#### 3.1.2. HPLC Analysis of BRP Extracts

**Figure 1:**
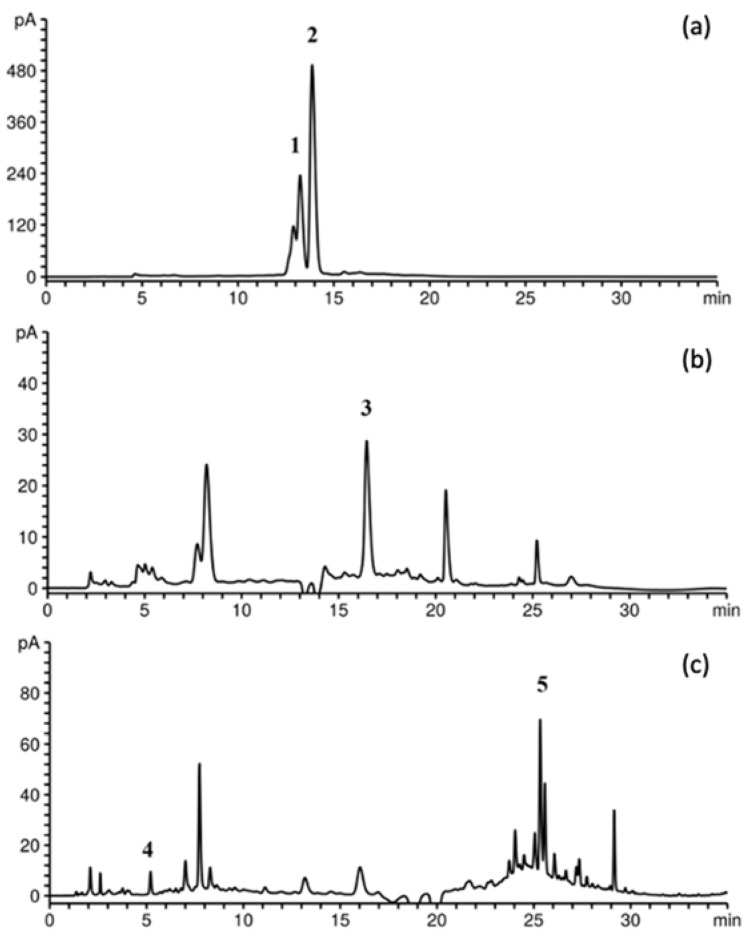
HPLC chromatograms of phenolic compounds from BRP extracts with detection at (**a**) 520 nm for 1-cyanidin-3-glucoside and 2-cyanidin-3-rutinoside, (**b**) 350 nm for 3-rutin, and (**c**) 260 nm for 4-gallic acid and 5-ellagic acid.

Raspberry is abundant in a specific class of phytochemicals, anthocyanins, which play a significant role in the majority of its biological effects and organoleptic attributes. The identification of principal anthocyanins and other phenolic compounds in BRP extracts was performed by HPLC-DAD, comparing their relative retention time and UV spectra with those of the standard solutions; the results are summarized in Figure 1 and Table 2. Five phenolic compounds were identified in BRP extracts, of which cyanidin-3-rutinoside was the main compound, followed by cyanidin-3-glucoside, rutin, gallic acid, and ellagic acid. These results are in accordance with the work of other authors, who also detected cyanidin-3-rutinoside as the main phenolic compound in BRP. Gil et al. [27] performed acidified methanol maceration for 1 h to obtain anthocyanins from raspberry pomace and noticed that cyanidins represented 95% of the total anthocyanins, and after cyanidin-3-rutinoside, the compounds at higher concentrations were cyanidin-3-sambubioside-5-rhamnoside and cyanidin-3-glucoside. In a study by Saad et al. [19], the analysis of raspberry pomace extracts obtained by aqueous enzyme-assisted extraction revealed the presence of gallic and ellagic acid. Rutin has been previously characterized in black raspberry fruit [28] and red raspberry pomace [4] but not in BRP. The findings obtained from the tentative HPLC analysis of the Chinese raspberry extracts revealed that gallic acid, rutin, and ellagic acid were identified together with quercetin 3-O-glucoside, avicularin, kaempferol-7-O-glucuronide, and quercetin-7-O-glucuronide [29]. 

From the results depicted in Table 2, it can be seen that the amounts of the two main constituents, cyanidin-3-glucoside and cyanidin-3-rutinoside, accounted for more than half of the total phenols (i.e., UAE10). The conventional extract (SLE1, 30% EtOH, 24 h) was found to contain 4.44 and 1.07 mg/g DW of cyanidin-3-rutinoside and cyanidin-3-glucoside, respectively, which was more than fourfold lower than that in the UAE extract obtained under optimal conditions (A 60%, 10 min). Under the same UAE conditions, the content of rutin was the highest (1.51 mg/g DW). UAE extracts contained an amount of cyanidin-3-rutinoside (11.98–17.50 mg/g DW) exceeding the yield obtained by Gil et al. [27] during acidified methanol maceration for 1 h (~3.6 mg/g pomace). A different trend in the content of the main compound was observed in the study of Szymanowska and Baraniak [30], in which they conveyed that the contents of cyanidin-3-glucoside and cyanidin-3-rutinoside were about 97.6 µg/g FW and 12.8 µg/g FW in pomace obtained from enzymatically treated raspberries. The gallic acid content was the highest in SLE1 and UAE1 extracts (0.18 and 0.13 mg/g DW), in both cases significantly surpassing the yields provided by UAE (UAE2-13), which was below 0.05 mg/g DW. The highest content of ellagic acid was observed in the SLE extract (0.77 mg/g DW), while UAE13 provided a slightly lower value (0.62 mg/g DW). Using acidic and alkali treatment, Yao et al. [4] obtained slightly lower extraction yields of rutin and gallic and ellagic acids for red raspberry pomace without seeds (1.86, 604.65, and 773.12 μg/g DW, respectively).

Few significant differences were observed between amplitude levels regarding extraction time. The contents of cyanidin-3-rutinoside, cyanidin-3-glucoside, and ellagic acid increased significantly with ultrasonic amplitude at a constant extraction time. Nevertheless, for the rutin and gallic acid contents, at certain times, there was a statistical difference between amplitudes, and at other times, there was not. Ultrasonic amplitude affects the compression and rarefaction cycle of ultrasonic waves, promoting interfacial turbulence and the release of the phenolic compounds from the matrix into the solvent [31]. It is possible to argue that ultrasonic waves cause the creation, growth, and eventual collapsing of microscopic bubbles, leading to the rupture of the cell wall of the BRP tissue and increasing the extraction rate. Moreover, it is worth noting that the temperature of the extraction medium increased in accordance with the applied amplitude. The findings of this study indicate that high-intensity ultrasound led to a significant increase in the extraction medium’s temperature, from 21 to 80 °C. In Table 2, it is possible to observe that there were no degradation effects on cyanidin-3-rutinoside, rutin, ellagic acid, or gallic acid at the highest amplitude (and consequently higher temperatures) and higher sonication time. However, the effects of high-intensity probe treatment, encompassing mechanical agitation, microjets, microtransmission, hot spots, and shock waves, were also observed by Xue et al. [32]. They noted that a higher extraction temperature had an adverse effect on the recovery of anthocyanins from raspberry wine residues. The recovery initially increased as the temperature increased from 40 to 50 °C, followed by a decrease at higher temperatures.

Although the amplitude of 60% during 10 min favored the recovery of principal phenolic compounds from BRP, the lower energy consumption with slightly lower phenolic yields was achieved at a higher amplitude and shorter extraction time (Table S2). Overall, UAE via sonotrode proved to be a fast (the minimum extraction time was 2 min) and efficient alternative with high-quality hydrophilic extracts using Generally Recognized as Safe solvents (30% EtOH), promoting/supporting the value addition and biorefinery as an agroindustrial by-product revalorization platform for the creation of a circular economy. Furthermore, the industrialization of this sono-based methodology in terms of its scale-up into continuous or large-scale processes might be a focus for future research.

### 3.2. Characterization of BRP Powder

#### 3.2.1. Encapsulation Yield and HPLC Analysis of BRP Powder

In a prior study, an optimization investigation was carried out to determine the optimal concentrations of various carriers, including maltodextrin, fenugreek gum, microcrystalline cellulose, agave fructans, and maltodextrin, in a dispersion formulation that was atomized for the production of black raspberry juice and raspberry juice powders using a spray dryer [33,34]. The red raspberry puree was encapsulated using a spray-drying method with gum arabic [35]. However, in order to minimize the potential degradation of anthocyanins caused by the high inlet/outlet temperatures in the spray-drying process, reduce expenses, and align with the principles of environmentally friendly processes, it might be beneficial to consider the application of the PGSS technique. These findings should be substantiated by a cost analysis that takes into account the influence of PGSS conditions.

Once the optimal extraction conditions were selected (SLE1 and UAE12), a high-pressure encapsulation process via supercritical CO_2_ was assessed. Recently, this technique has found applications in the microencapsulation of linseed oil enriched with carrot pomace [11], brown algae pigments [36], cinnamon and paprika oleoresins [37], and oils from brewer’s spent grain [38]. However, the PGSS efficiency for polyphenols from BRP extracts has never been evaluated. PGSS with UAE extracts was found to attain higher EnY compared with the values of the SLE ones, regardless of the pressure and the extract-to-carrier ratio applied (Table 3). Furthermore, the effect of the applied pressure and the extract-to-carrier ratio in the PGSS reactor was investigated with regard to the powder properties. It was observed that the values for microparticles generated at 100 bar were significantly lower than those obtained at 200 and 300 bar. An increase from 100 to 200 bar decreased the EnY from 7 to 34% depending on the encapsulated powder. Regarding the impact of the BRP extract-to-carrier ratio, the lower content of GMS resulted in a lower EnY. The highest EnYs resulted in microparticles obtained at 150 and 200 bar with the SLE (E2, 73.49%) and UAE (E3, 66.76%) extracts, respectively, at a ratio of 1:11.

**Figure 2:**
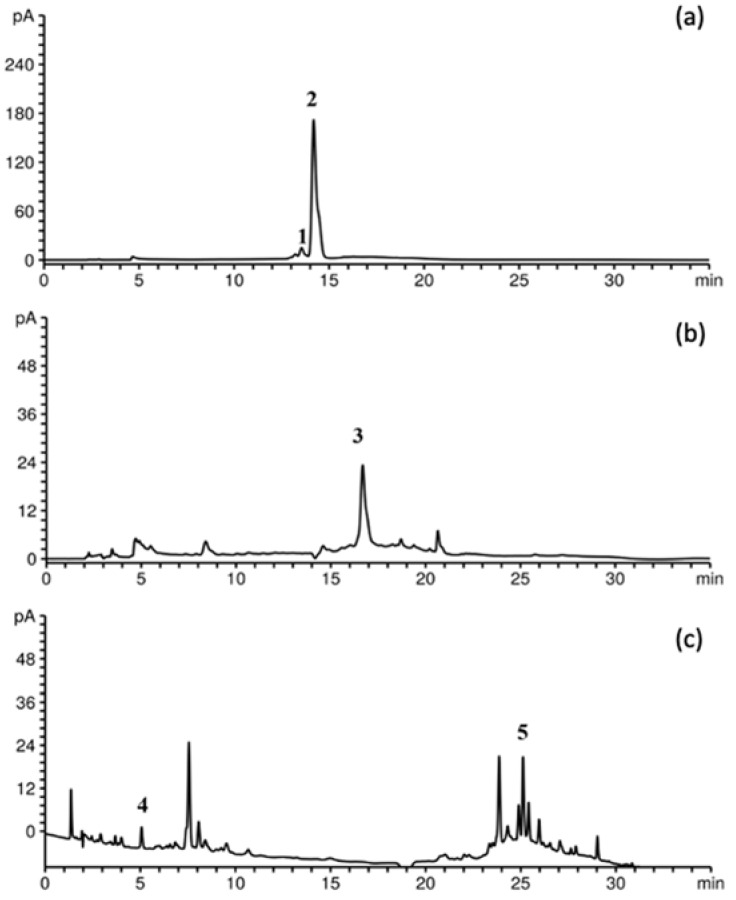
HPLC chromatogram of identified phenolics in BRP powder recorded at (**a**) 520 nm for 1-cyanidin-3-glucoside and 2-cyanidin-3-rutinoside, (**b**) 350 nm for 3-rutin, and (**c**) 260 nm for 4-gallic acid and 5-ellagic acid.

The individual phenolic compounds of the BRP in the resulting powder were determined by HPLC-DAD (Figure 2). As in the BRP extracts, cyanidin-3-O-rutinoside was the main compound, accounting for 11.71 mg/g of the powder (SLE E7), followed by rutin (UAE E8, 0.91 mg/g), cyanidin-3-O-glucoside (SLE E6, 0.85 mg/g), ellagic acid (SLE E2, 0.32 mg/g), and gallic acid (UAE E8, 0.1 mg/g). Cyanidin-3-O-rutinoside, a natural antioxidant, proved to have protective effects on various tissues and cell types. Chen et al. [39] reported the inhibitory effect of cyanidin 3-O-rutinoside on the migration of human lung cancer cell lines. Cyanidin-3-O-rutinoside also demonstrated the ability to inhibit cyclooxygenase enzymes, which play a crucial role in processes such as inflammation, carcinogenesis, apoptosis, cell proliferation, and angiogenesis [40].

For cyanidin-3-O-rutinoside, cyanidin-3-O-glucoside, and ellagic acid, the SLE powders exhibited the highest contents in comparison to the UAE ones, while gallic acid was quantified only in the UAE powders. UAE also showed mild enhancement of rutin content in comparison with SLE. It was apparent that the contents of individual phenolics in the BRP powders were positively influenced by the extract-to-carrier ratio at the same pressure, and they were the highest in the BRP powders with the highest content of the extract. By decreasing the content of the carrier from 2.50 to 2.25 g with SLE extract and at a pressure of 200 bar, its ability to protect only cyanidin-3-O-glucoside decreased by 50%, which was not in line with the previous trend. On the other hand, at the same extract-to-carrier ratio, there was no commensurate increase or decrease in the phenolic content with a change in pressure. The highest contents of rutin, ellagic acid, and gallic acid were measured in the BRP powder obtained at 150 bar, while encapsulation at pressures of 100 and 200 bar proved to have better recoveries of cyanidin-3-O-rutinoside and cyanidin-3-O-glucoside, respectively. However, we cannot conclude which type of extract, extract-to-carrier ratio, and pressure are optimal because each combination of conditions appears to be more adequate for the encapsulation of each individual compound.

#### 3.2.2. Particles Size, Bulk and Tapped Density, and Morphology of BRP Powder

The particle size of the powdered extracts was affected by the liquid feed (material to be encapsulated) composition and viscosity, the applied carrier type and concentration, and the encapsulation technology applied (Table 4). In the study, liquid feeds contained ethanolic BRP extracts, which were obtained through two different methods (SLE and UAE). According to the results, these extracts showed differences in the extract composition and the profile of bioactive constituents measured. Furthermore, the concentration of the applied carrier material was different. Still, it seems that the difference in the liquid feed composition did not notably affect the particle size of the obtained powders. Regarding the effect of the technology applied, according to the obtained results, the pressure applied in PGSS also did not show a notable impact on the powder’s particle size. PGSS encapsulation resulted in quite small particles of micro-size, in the range of 4.87 to 6.26 μm for the SLE powder and in a narrower range in the case of the UAE powder, from 4.85 to 5.51 μm. The particle size of the powders is of importance as it significantly affects the physical properties of the powders as well as their possible performance, applications, and effects. Namely, powders with a reduced particle size are more desirable, as when ingested orally, they can increase the solubility and bioavailability of the bioactive ingredients, leading to their increased absorption in the organism and improved efficiency. Furthermore, in the final products, such as cosmetic ones, powders with reduced particle size are also desired because they enable the formation of a more adequate, smooth product texture. From this point of view, the obtained BRP powders could be of potential value for pharmaceutical and cosmetic product applications.

The production of powders of appropriate bulk and tapped density is also of importance, especially in the pharmaceutical industry, in which powder is intended to be used for the creation of limited-volume dosage forms, such as pills and tablets. Therefore, a higher tapped and bulk density is desired, as it enables the creation of dosage forms with an increased concentration of active ingredients. From this point of view, in the case of the SLE powders, a higher bulk and tapped density were achieved in the SLE1, SLE8, and SLE9 powders, while in the case of the UAE powders, this was obtained in UAE E1-3 and UAE E7.

Interparticle interactions that impact the bulk properties of a powder are essentially interactions that hinder the flow of the powder. Therefore, by comparing the bulk density to the tapped density, it is possible to assess the significance of these interactions in a particular powder. This comparison can serve as an indicator of the powder’s flowability. For materials that flow poorly, there are stronger interparticle interactions, leading to a notable difference between the bulk and tapped densities. This difference is quantified in terms of the Carr index. The flow characteristics of the BRP powders were evaluated by determining their bulk and tapped densities. Subsequently, the Carr index and Hausner ratio were computed to assess these flow characteristics (Table 4). According to the obtained results of the Carr index, for all powders, this index was above 15%, meaning that there were no powders with excellent flow properties. Thus, several powders possessed acceptable characteristics, as their Carr index was up to 25%. These were SLE E1, SLE E3, SLE E4, SLE E5, and SLE E7, and only one UAE powder—E4. Regarding the Hausner index, for almost all powders, this value was in the range of 1.25 to 1.5, meaning that almost all, except UAE E1, UAE E8, and UAE E9, had acceptable values, but again, no powders could be characterized as having excellent flow properties. The powder with the most appropriate flow characteristics was UAE E5, for which the Carr index was 20% and the Hausner index was 1.25. However, this was the powder with the lowest bulk and tapped density, and this excluded it from being recommended as the most appropriate to be used. Considering all four characteristics—the tapped and bulk density and the Carr and Hausner indexes—only one BRP powder could be selected as the powder with the desired characteristics, and this was SLE E1. Regarding the impact of the liquid feed or pressure applied in PGSS, no certain or exact pattern could be applied for the explanation of the difference in these powders’ flow characteristics. Because of the powder’s flow properties, which were mostly in the range of poor to acceptable, the application and usage of this kind of powder may induce a certain cohesion between particles, leading to problems during application on an industrial level, such as segregation or fluctuation in the flow rate. One of the possible solutions for the improvement of the obtained powder’s flowability could be the addition of an adequate carrier that could improve this powder’s characteristics. The selection of a carrier for this purpose, of course, depends on the final product’s desired form and application.

**Figure 3:**
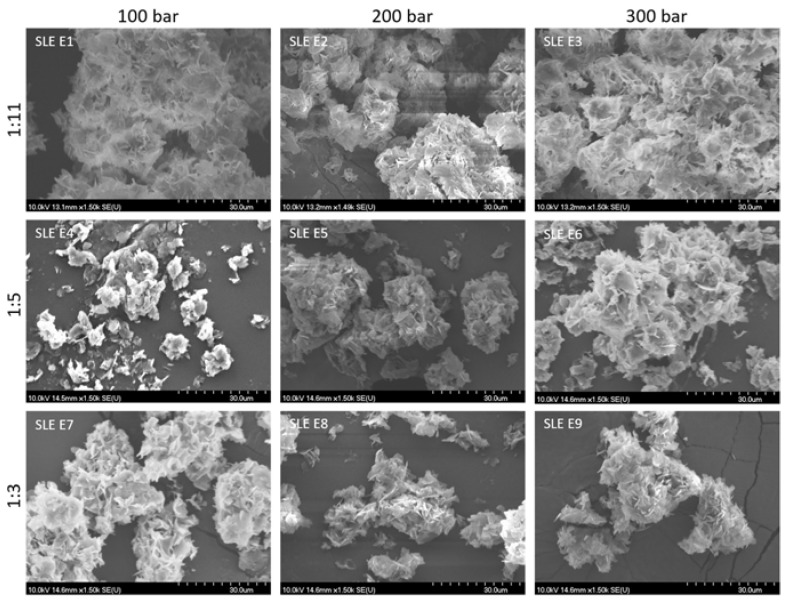
SEM images of BRP powders obtained after SLE extraction.

**Figure 4:**
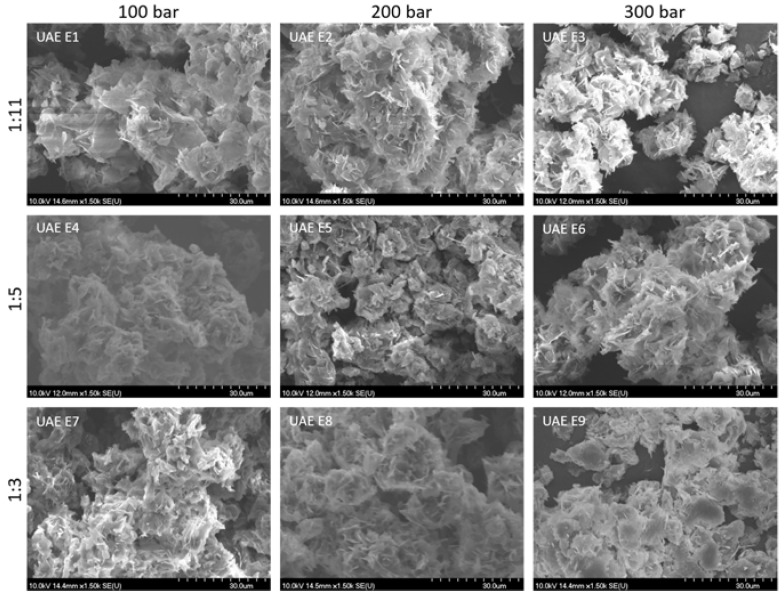
SEM images of BRP powders obtained after UAE extraction.

As shown in the microscopic pictures (Figure 3 and Figure 4), the thin sharp plate form was present in the powders instead of the spherical form, which are characteristics of other encapsulated techniques, such as spray drying. These thin sharp plate forms showed aggregation formations in all encapsulated particles. There were differences in the aggregation density depending on how the thin sharp plate forms were organized. Formed aggregates were more intensive and larger in the case of the SLE powders obtained with a range of carrier of 1:11 and in the case of all UAE powders. In the case of these aggregates, their size was wider than 60 µm. The existence of large aggregates instead of individual dispersed encapsulated particles may impact the powder’s solubility and efficiency. The surface texture of the aggregates was dominantly rough and sharp, with a certain low amount of a smooth, near-spherical surface noticed only in the case of UAE E8 and E9 and SLE E1-3 and E6. The presence of pores or cracks on the thin plate forms did not exist. Generally, the absence of particles of the uniform spherical form might indicate that the encapsulated bioactive compounds are distributed and localized on the entire thin plate surface, allowing increased contact with external stress factors and eventually leading to lower stability or the disintegration of the encapsulated constituents.

## 4. Conclusions

Two sustainable techniques, sonotrode-based extraction and the supercritical CO_2_ encapsulation process, were applied to produce a high-quality powder. In the first step, UAEs with different amplitudes and sonication times were evaluated. The sonication amplitude strongly affected UAE, in which the highest amplitude (100%) and moderate extraction time (4/6 min) provided higher yields and the TPC, while lower amplitude (60%) favored the recovery of principal anthocyanins, cyanidin-3-rutinoside, and cyanidin-3-glucoside. Five phenolic compounds were identified in the BRP extracts by HPLC, of which cyanidin-3-rutinoside was the main compound, followed by cyanidin-3-glucoside, rutin, gallic acid, and ellagic acid. The choice of the optimal extraction conditions in the UAE (A = 100%, t = 4 min) was made according to the influence of the process parameters in terms of overall extraction yield, total phenolics, specific phenolics, and energy expenditure. In the second step, PGSS using GMS as a carrier formulated an acceptable form of BRP extract. The main phenolic compound in the BRP powders was cyanidin-3-rutinoside, following the same trend as BRP extracts. The contents of individual phenolics in the BRP powders were the highest in those with the highest content of extract (0.75 g). A lower extract-to-carrier ratio positively influenced particle size, bulk and tapped density, and Carr and Hausner indexes of the BRP powders. This work is the first to produce anthocyanin-rich fractions from industrial BRP using such time-efficient and high-performance techniques, offering insights for further research and industrial scale-up into continuous or large-scale processes. In addition, future studies should focus on the application of other carriers as health-promoting ingredients and the determination of in vitro bioaccessability of the obtained PGSS powder.

## Figures and Tables

**Table 1:** EYs and TPCs obtained by SLE and UAE of BRP.

Sample Code	Extraction Conditions	EY (%) *	TPC (mg GAE/g DW) *
SLE1	30% EtOH	16.46 ± 0.97 ^a^	37.97 ± 3.28 ^a^
SLE2	50% EtOH	15.66 ± 0.68 ^a^	34.35 ± 0.78 ^b^
SLE3	70% EtOH	15.37 ± 0.81 ^a^	31.51 ± 0.90 ^b^
UAE1	A 20%, 2 min, 34.5 °C	10.77 ± 0.53^d^	24.22 ± 1.01 ^h^
UAE2	A 20%, 4 min, 37.5 °C	14.68 ± 0.22 ^abc^	27.44 ± 0.34 ^g^
UAE3	A 20%, 6 min, 39 °C	13.58 ± 0.62 ^c^	23.29 ± 0.56 ^h^
UAE4	A 20%, 8 min, 42.5 °C	13.85 ± 0.33 ^bc^	31.04 ± 1.36 ^ef^
UAE5	A 20%, 10 min, 45.5 °C	15.42 ± 0.81 ^ab^	39.54 ± 1.20 ^c^
UAE6	A 60%, 2 min, 43.5 °C	14.64 ± 1.08 ^abc^	29.51 ± 0.53 ^fg^
UAE7	A 60%, 4 min, 51.5 °C	13.48 ± 0.76 ^c^	32.65 ± 0.75 ^de^
UAE8	A 60%, 6 min, 62.5 °C	15.25 ± 0.03 ^abc^	34.34 ± 0.28 ^d^
UAE9	A 60%, 8 min, 65 °C	15.22 ± 0.68 ^abc^	40.25 ± 0.51 ^c^
UAE10	A 60%, 10 min, 70 °C	16.03 ± 0.49 ^a^	42.42 ± 0.55 ^b^
UAE11	A 100%, 2 min, 52 °C	13.96 ± 0.20 ^bc^	32.31 ± 0.62 ^de^
UAE12	A 100%, 4 min, 67.5 °C	15.77 ± 0.73 ^a^	44.74 ± 0.11 ^a^
UAE13	A 100%, 6 min, 80 °C	16.09 ± 0.57 ^a^	41.64 ± 0.52 ^bc^

* Different letters within a column indicate significant differences between samples at *p* < 0.05.

**Table 2:** Contents of the identified phenolic compounds detected by HPLC-DAD in BRP extracts obtained by SLE and UAE.

Samples	Mean ± SD (mg/g DW; n = 3) *				
	C3Rut	C3Glu	RUT	GA	EA
SLE1	4.4443 ± 0.1686 ^i^	1.0768 ± 0.0226 ^i^	0.9497 ± 0.0045 ^c^	0.1835 ± 0.0042 ^a^	0.7653 ± 0.0180 ^a^
UAE1	12.3115 ± 0.0635 ^gh^	3.6032 ± 0.1148 ^fgh^	0.6711 ± 0.0260 ^e^	0.1257 ± 0.0039 ^b^	0.3083 ± 0.0051 ^h^
UAE2	11.9834 ± 0.4970 ^h^	3.3658 ± 0.0681 ^h^	0.6886 ± 0.0091 ^e^	0.0406 ± 0.0010 ^c^	0.3276 ± 0.0022 ^h^
UAE3	12.4663 ± 0.3485 ^gh^	3.4983 ± 0.0771 ^gh^	0.7018 ± 0.0113 ^e^	0.0365 ± 0.0010 ^d^	0.4046 ± 0.0072 ^g^
UAE4	13.1566 ± 0.2001 ^gh^	3.7791 ± 0.0661 ^efg^	0.8572 ± 0.0286 ^d^	0.0236 ± 0.0005 ^f^	0.4917 ± 0.0187 ^de^
UAE5	16.2183 ± 0.1689 ^bc^	4.6325 ± 0.1299 ^ab^	0.9707 ± 0.0203 ^c^	0.0298 ± 0.0005 ^e^	0.4559 ± 0.0039 ^ef^
UAE6	14.5262 ± 0.0143 ^ef^	3.7119 ± 0.0373 ^efg^	0.9345 ± 0.0334 ^cd^	0.0271 ± 0.0011 ^ef^	0.4244 ± 0.0195 ^fg^
UAE7	16.9502 ± 0.1629 ^ab^	4.6577 ± 0.1247 ^ab^	1.1320 ± 0.0273 ^b^	0.0122 ± 0.0003 ^h^	0.4914 ± 0.0174 ^de^
UAE8	14.7056 ± 0.3143 ^de^	4.0405 ± 0.1633 ^de^	1.0933 ± 0.0523 ^b^	0.0168 ± 0.0001 ^g^	0.5131 ± 0.0060 ^d^
UAE9	13.4015 ± 0.2345 ^fg^	3.2963 ± 0.0273 ^h^	1.4945 ± 0.0322 ^a^	0.0237 ± 0.0003 ^f^	0.5589 ± 0.0272 ^c^
UAE10	17.5031 ± 0.5750 ^a^	4.8878 ± 0.1772 ^a^	1.5102 ± 0.0582 ^a^	0.0309 ± 0.0012 ^e^	0.5931 ± 0.0050 ^bc^
UAE11	15.8023 ± 0.7863 ^bcd^	4.3972 ± 0.0034 ^bc^	0.6711 ± 0.0085 ^e^	0.0182 ± 0.0005 ^g^	0.4706 ± 0.0001 ^de^
UAE12	16.7786 ± 0.5246 ^ab^	4.1594 ± 0.1770 ^cd^	0.6886 ± 0.0285 ^e^	0.0185 ± 0.0008 ^g^	0.5854 ± 0.0195 ^bc^
UAE13	15.0331 ± 0.7402 ^cde^	3.8538 ± 0.1177 ^def^	0.7018 ± 0.0168 ^e^	0.0369 ± 0.0007 ^cd^	0.6206 ± 0.0270 ^b^

* C3Rut—cyanidin-3-O-rutinoside, C3Glu—cyanidin-3-O-glucoside, RUT—rutin, GA—gallic acid, EA—ellagic acid. Different letters within a column indicate significant differences between samples at *p* < 0.05.

**Table 3:** EnYs and content of the identified phenolic compounds detected by HPLC-DAD in BRP powders.

Sample	Encapsulation Conditions (Pressure, Mass Ratio of BRP Extract to Carrier)	EnY (%)	Mean ± SD (mg/g Powder; n = 3) *				
			C3Rut	C3Glu	RUT	GA	EA
SLE E1	100 bar, 1:11	39.49 ± 2.89 ^ef^	3.7115 ± 0.1361 ^hi^	0.1474 ± 0.0073 ^ijk^	0.4784 ± 0.0175 ^f^	n.d.	0.0783 ± 0.0037 ^ef^
SLE E2	150 bar, 1:11	73.49 ± 5.81 ^a^	2.7786 ± 0.1027 ^j^	0.1128 ± 0.0006 ^m^	0.3559 ± 0.0036 ^h^	n.d.	0.0326 ± 0.0006 ^i^
SLE E3	200 bar, 1:11	60.07 ± 4.60 ^abc^	3.5096 ± 0.0886 ^i^	0.1352 ± 0.0006 ^jkl^	0.3876 ± 0.0188 ^gh^	n.d.	0.0526 ± 0.0025 ^gh^
SLE E4	100 bar, 1:5	51.03 ± 3.84 ^b–e^	6.1310 ± 0.3031 ^e^	0.2403 ± 0.0000 ^f^	0.4811 ± 0.0205 ^f^	n.d.	0.0708 ± 0.0016 ^f^
SLE E5	150 bar, 1:5	58.19 ± 8.55 ^a–d^	10.5370 ± 0.1600 ^b^	0.5203 ± 0.0070 ^d^	0.6629 ± 0.0158 ^d^	n.d.	0.0663 ± 0.0012 ^fg^
SLE E6	200 bar, 1:5	66.72 ± 7.56 ^ab^	9.1159 ± 0.2094 ^d^	0.8530 ± 0.0044 ^a^	0.5827 ± 0.0130 ^e^	n.d.	0.0624 ± 0.0007 ^fg^
SLE E7	100 bar, 1:3	27.11 ± 4.00 ^f^	11.7135 ± 0.5560 ^a^	0.6612 ± 0.0133 ^b^	0.8397 ± 0.0277 ^bc^	n.d.	0.1577 ± 0.0058 ^d^
SLE E8	150 bar, 1:3	41.35 ± 4.17 ^def^	10.7481 ± 0.2510 ^b^	0.5447 ± 0.0122 ^c^	0.8847 ± 0.0015 ^ab^	n.d.	0.3226 ± 0.0134 ^a^
SLE E9	200 bar, 1:3	49.23 ± 7.06 ^cde^	9.9409 ± 0.0502 ^c^	0.4257 ± 0.0033 ^e^	0.8068 ± 0.0354 ^c^	n.d.	0.1794 ± 0.0084 ^c^
UAE E1	100 bar, 1:11	49.16 ± 2.57 ^cde^	1.2185 ± 0.0185 ^k^	0.1318 ± 0.0064 ^klm^	0.2931 ± 0.0085 ^i^	n.d.	/
UAE E2	150 bar, 1:11	66.49 ± 8.17 ^ab^	2.2137 ± 0.010^0 j^	0.1217 ± 0.0040l ^m^	0.2949 ± 0.0003 ^i^	n.d.	0.0420 ± 0.0013 ^hi^
UAE E3	200 bar, 1:11	66.76 ± 3.56 ^ab^	0.6274 ± 0.0022 ^l^	0.0379 ± 0.0002 ^n^	0.0815 ± 0.0001 ^j^	n.d.	0.0391 ± 0.0005 ^hi^
UAE E4	100 bar, 1:5	64.47 ± 7.70 ^abc^	4.2728 ± 0.1986 ^gh^	0.1566 ± 0.0045 ^hi^	0.5820 ± 0.0220 ^e^	0.0120 ± 0.0005d	0.0403 ± 0.0013 ^hi^
UAE E5	150 bar, 1:5	63.16 ± 4.49 ^abc^	4.2487 ± 0.0955 ^gh^	0.1717 ± 0.0081 ^h^	0.4200 ± 0.0057 ^g^	0.0300 ± 0.0014c	0.1430 ± 0.0040 ^d^
UAE E6	200 bar, 1:5	56.03 ± 4.28 ^b–e^	4.3270 ± 0.1812 ^g^	0.1677 ± 0.0046 ^h^	0.8801 ± 0.0287 ^ab^	0.0620 ± 0.0028b	0.0935 ± 0.0030 ^e^
UAE E7	100 bar, 1:3	29.57 ± 4.39 ^f^	4.3873 ± 0.0201 ^g^	0.1536 ± 0.0032 ^hij^	0.6539 ± 0.0147 ^d^	0.0580 ± 0.0020b	0.2949 ± 0.0120 ^b^
UAE E8	150 bar, 1:3	52.98 ± 3.43 ^b–e^	5.0181 ± 0.0779 ^f^	0.1940 ± 0.0023 ^g^	0.9116 ± 0.0132 ^a^	0.0950 ± 0.0038a	0.2980 ± 0.0118 ^b^
UAE E9	200 bar, 1:3	52.19 ± 7.35 ^b–e^	4.4735 ± 0.0256 ^fg^	0.5119 ± 0.0111 ^d^	0.5871 ± 0.0235 ^e^	n.d.	0.0747 ± 0.0032 ^f^

* C3Rut—cyanidin-3-O-rutinoside, C3Glu—cyanidin-3-O-glucoside, RUT—rutin, GA—gallic acid, EA—ellagic acid. Different letters within a column indicate significant differences between samples at *p* < 0.05; n.d.—not detected.

**Table 4:** Mean particle size and bulk and tapped density of BRP powders.

Sample	Particle Size (µm)	Bulk Density (g/mL)	Tapped Density (g/mL)	Carr Index (%)	Hausner Ratio	Flow Character
SLE E1	5.83 ± 0.99	0.180	0.240	25	1.33	Acceptable
SLE E2	5.82 ± 3.04	0.063	0.090	30	1.43	Poor
SLE E3	6.02 ± 1.09	0.058	0.074	22	1.28	Acceptable
SLE E4	6.23 ± 1.12	0.102	0.130	22	1.27	Acceptable
SLE E5	6.2 ± 1.05	0.068	0.091	25	1.34	Acceptable
SLE E6	6.26 ± 2.84	0.068	0.100	32	1.47	Poor
SLE E7	6.06 ± 1.23	0.066	0.086	23	1.30	Acceptable
SLE E8	4.87 ± 0.62	0.206	0.302	32	1.47	Poor
SLE E9	5.06 ± 0.69	0.200	0.277	28	1.39	Poor
UAE E1	5.46 ± 0.84	0.188	0.284	34	1.51	Poor
UAE E2	5.54 ± 0.95	0.200	0.270	26	1.35	Poor
UAE E3	5.51 ± 0.85	0.208	0.288	28	1.38	Poor
UAE E4	4.97 ± 0.68	0.052	0.078	33	1.50	Poor
UAE E5	4.85 ± 0.59	0.072	0.090	20	1.25	Acceptable
UAE E6	5.33 ± 0.86	0.054	0.079	32	1.46	Poor
UAE E7	5.44 ± 0.94	0.222	0.300	26	1.35	Poor
UAE E8	5.35 ± 0.86	0.048	0.075	36	1.56	Poor
UAE E9	5.29 ± 0.79	0.052	0.081	36	1.56	Poor

## Data Availability

The data used to support the findings of this study can be made available by the corresponding author upon request.

## Supplementary Materials

The following supporting information can be downloaded at: https://www.mdpi.com/article/10.3390/foods12203867/s1, Table S1: Temperature variation in the UAE from BRP; Table S2: Energy variation in the UAE from BRP.

## References

[1] Jokić S., Nastić N., Vidović S., Flanjak I., Aladić K., Vladić J. (2020). An Approach to Value Cocoa Bean By-Product Based on Subcritical Water Extraction and Spray Drying Using Different Carriers. Sustainability.

[2] Rashid R., Masoodi F.A., Wani S.M., Manzoor S., Gull A. (2020). Ultrasound assisted extraction of bioactive compounds from pomegranate peel, their nanoencapsulation and application for improvement in shelf life extension of edible oils. Food Chem..

[3] Tubiello F.N., Rosenzweig C., Conchedda G., Karl K., Gütschow J., Xueyao P., Obli-Laryea G., Wanner N., Qiu S.Y., Barros J.D. (2021). Greenhouse gas emissions from food systems: Building the evidence base. Enviro. Res. Lett..

[4] Yao J., Chen J., Yang J., Hao Y., Fan Y., Wang C., Li N. (2021). Free, soluble-bound and insoluble-bound phenolics and their bioactivity in raspberry pomace. LWT.

[5] Li M., Liu Y., Yang G., Sun L., Song X., Chen Q., Bao Y., Luo T., Wang J. (2022). Microstructure, physicochemical properties, and adsorption capacity of deoiled red raspberry pomace and its total dietary fiber. LWT.

[6] Četojević-Simin D.D., Velićanski A.S., Cvetković D.D., Markov S.L., Ćetković G.S., Tumbas Šaponjac V.T., Vulić J.J., Čanadanović-Brunet J.M., Djilas S.M. (2015). Bioactivity of Meeker and Willamette raspberry (*Rubus idaeus* L.) pomace extracts. Food Chem..

[7] Mutavski Z., Nastić N., Živković J., Šavikin K., Veberič R., Medič A., Pastor K., Jokić S., Vidović S. (2022). Black Elderberry Press Cake as a Source of Bioactive Ingredients Using Green-Based Extraction Approaches. Biology.

[8] (2022). FAOSTAT. https://www.fao.org/faostat/en/#data/QCL/visualize.

[9] Melgosa R., Sanz M.T., Beltrán S. (2021). Supercritical CO_2_ processing of omega-3 polyunsaturated fatty acids–Towards a biorefinery for fish waste valorization. J. Supercrit. Fluids.

[10] Li J., Pettinato M., Casazza A.A., Perego P. (2022). A comprehensive optimization of ultrasound-assisted extraction for lycopene recovery from tomato waste and encapsulation by spray drying. Processes.

[11] Klettenhammer S., Ferrentino G., Zendehbad H.S., Morozova K., Scampicchio M. (2022). Microencapsulation of linseed oil enriched with carrot pomace extracts using Particles from Gas Saturated Solutions (PGSS) process. J. Food Eng..

[12] Bamba B.S.B., Shi J., Tranchant C.C., Xue S.J., Forney C.F., Lim L.T. (2018). Influence of extraction conditions on ultrasound-assisted recovery of bioactive phenolics from blueberry pomace and their antioxidant activity. Molecules.

[13] Kobus Z., Krzywicka M., Pecyna A., Buczaj A. (2021). Process efficiency and energy consumption during the ultrasound-assisted extraction of bioactive substances from hawthorn berries. Energies.

[14] Krivošija S., Jerković I., Nastić N., Zloh M., Jokić S., Banožić M., Aladić K., Vidović S. (2023). Green pathway for utilisation of orange peel dust and in silico evaluation of pharmacological potential. Microchem. J..

[15] Singleton V.L., Rossi J.A. (1965). Colorimetry of total phenolics with phosphomolybdic-phosphotungstic acid reagents. Am. J. Enol. Vitic..

[16] Carr R.L. (1965). Evaluating flow properties of solids. Chem. Eng..

[17] Živković J., Vladić J., Naffati A., Nastić N., Šavikin K., Tomić M., Vidović S. (2022). Comparative Chemical Profiling of Underexploited *Arctostaphylos uva-ursi* L. Herbal Dust Extracts Obtained by Conventional, Ultrasound-Assisted and Subcritical Water Extractions. Waste Biomass Valori..

[18] Kumar K., Srivastav S., Sharanagat V.S. (2021). Ultrasound assisted extraction (UAE) of bioactive compounds from fruit and vegetable processing by-products: A review. Ultrason. Sonochem..

[19] Oliveira A.M.B., Viganó J., Sanches V.L., Rostagno M.A., Martínez J. (2022). Extraction of potential bioactive compounds from industrial Tahiti lime (*Citrus latifólia* Tan.) by-product using pressurized liquids and ultrasound-assisted extraction. Food Res. Int..

[20] Rocha R., Pinela J., Abreu R.M.V., Añibarro-Ortega M., Pires T.C.S.P., Saldanha A.L., Alves M.J., Nogueira A., Ferreira I.C.F.R., Barros L. (2020). Extraction of Anthocyanins from Red Raspberry for Natural Food Colorants Development: Processes Optimization and In Vitro Bioactivity. Processes.

[21] Boateng I.D., Kumar R., Daubert C.R., Flint-Garcia S., Mustapha A., Kuehnel L., Agliata J., Li Q., Wan C., Somavat P. (2023). Sonoprocessing improves phenolics profile, antioxidant capacity, structure, and product qualities of purple corn pericarp extract. Ultrason. Sonochem..

[22] Kryževičiūtė N., Kraujalis P., Venskutonis P.R. (2016). Optimization of high pressure extraction processes for the separation of raspberry pomace into lipophilic and hydrophilic fractions. J. Supercrit. Fluids.

[23] Krivokapić S., Vlaović M., Damjanović Vratnica B., Perović A., Perović S. (2021). Biowaste as a Potential Source of Bioactive Compounds—A Case Study of Raspberry Fruit Pomace. Foods.

[24] Saad N., Louvet F., Tarrade S., Meudec E., Grenier K., Landolt C., Ouk T.S., Bressollier P. (2019). Enzyme-Assisted Extraction of Bioactive Compounds from Raspberry (*Rubus idaeus* L.) Pomace. J. Food Sci..

[25] Park S.J., Hyun S.-H., Suh H.W., Lee S.-Y., Min T.-S., Auh J.-H., Lee H.-J., Kim J.-H., Cho S.-M., Choi H.-K. (2012). Differentiation of black raspberry fruits according to species and geographic origins by genomic analysis and 1H-NMR-based metabolic profiling. J. Korean Soc. Appl. Biol. Chem..

[26] Mazur S.P., Sønsteby A., Nes A., Wold A.B., Foito A., Freitag S., Verrall S., Stewart D., Heide O.M. (2014). Effects of post-flowering environmental variation along an altitudinal gradient on chemical composition of ‘Glen Ample’red raspberry (*Rubus idaeus* L.). Eur. J. Hortic. Sci..

[27] Gil A.S.R. (2013). Potential influence of blueberry and black raspberry pomace phenolics on inflammatory cytokines in coronary cells. Doctoral Thesis.

[28] Shin D., Chae K.S., Choi H.R., Lee S.J., Gim S.W., Kwon G.T., Lee H.T., Song Y.C., Kim K.J., Kong H.S. (2018). Bioactive and pharmacokinetic characteristics of pre-matured black raspberry *Rubus occidentalis*. Ital. J. Food Sci..

[29] Su J., Jin L., Yang R., Liang Y., Nile S.H., Kai G. (2023). Comparative studies on selection of high polyphenolic containing Chinese raspberry for evaluation of antioxidant and cytotoxic potentials. J. Agric Food Res..

[30] Szymanowska U., Baraniak B. (2019). Antioxidant and Potentially Anti-Inflammatory Activity of Anthocyanin Fractions from Pomace Obtained from Enzymatically Treated Raspberries. Antioxidants.

[31] Oroian M., Ursachi F., Dranca F. (2020). Influence of ultrasonic amplitude, temperature, time and solvent concentration on bioactive compounds extraction from propolis. Ultrason. Sonochem..

[32] Xue H., Tan J., Li Q., Tang J., Cai X. (2021). Ultrasound-Assisted Enzymatic Extraction of Anthocyanins from Raspberry Wine Residues: Process Optimization, Isolation, Purification, and Bioactivity Determination. Food Anal. Methods.

[33] Yousefi S., Emam-Djomeh Z., Mousavi M., Kobarfard F., Zbicinski I. (2015). Developing spray-dried powders containing anthocyanins of black raspberry juice encapsulated based on fenugreek gum. Adv. Powder Technol..

[34] Farías-Cervantes V.S., Salinas-Moreno Y., Chávez-Rodríguez A., Luna-Solano G., Medrano-Roldan H., Andrade-González I. (2020). Stickiness and agglomeration of blackberry and raspberry spray dried juices using agave fructans and maltodextrin as carrier agents. Czech J. Food Sci..

[35] Syamaladevi R.M., Insan S.K., Dhawan S., Andrews P., Sablani S.S. (2012). Physicochemical Properties of Encapsulated Red Raspberry (*Rubus idaeus*) Powder: Influence of High-Pressure Homogenization. Dry. Technol..

[36] Banožić M., Čolnik M., Škerget M., Cikoš A.-M., Aladić K., Jokić S. (2022). Formation and Characterization of *Fucus virsoides* J. Agardh Pigment–Polyethylene Glycol Microparticles Produced Using PGSS Process. Appl. Sci..

[37] Procopio F.R., Klettenhammer S., Ferrentino G., Scampicchio M., Do Amaral Sobral P.J., Hubinger M.D. (2023). Comparative Study of Cinnamon and Paprika Oleoresins Encapsulated by Spray Chilling and Particles from Gas Saturated Solutions Techniques: Evaluation of Physical Characteristics and Oleoresins Release in Food Simulated Media. Food Bioprocess Technol..

[38] Ndayishimiye J., Ferrentino G., Nabil H., Scampicchio M. (2020). Encapsulation of Oils Recovered from brewer’s Spent Grain by Particles from Gas Saturated Solutions Technique. Food Bioprocess Technol..

[39] Chen P.-N., Chu S.-C., Chiou H.-L., Kuo W.-H., Chiang C.-L., Hsieh Y.-S. (2006). Mulberry anthocyanins, cyanidin 3-rutinoside and cyanidin 3-glucoside, exhibited an inhibitory effect on the migration and invasion of a human lung cancer cell line. Cancer Lett..

[40] Mulabagal V., Lang G.A., DeWitt D.L., Dalavoy S.S., Nair M.G. (2009). Anthocyanin Content, Lipid Peroxidation and Cyclooxygenase Enzyme Inhibitory Activities of Sweet and Sour Cherries. J. Agric. Food Chem..

