# Supplementary material for: Green Processing of Black Raspberry Pomace: Application of Sonotrode-Based Extraction Technique and Particles from Gas-Saturated Solutions (PGSS) Technology
Source: Foods. 2023 Oct 22;12(20):3867. doi: 10.3390/foods12203867 (PMC10606185; doi:10.3390/foods12203867)
Supplement: Supplementary file 1 [file foods-12-03867-s001.zip › foods-2673291-supplementary.pdf]

SUPPLEMENTARY MATERIAL

Table S1. Temperature variation in the UAE from BRP.

t (min)	A (%)	ΔT (°C)
2	20	34.5 ± 0.7
2	60	43.5 ± 2.1
2	100	52.0 ± 0.0
4	20	37.5 ± 0.7
4	60	51.5 ± 3.5
4	100	67.5 ± 0.7
6	20	39.0 ± 0.0
6	60	61.5 ± 0.7
6	100	80.0 ± 0.0
8	20	42.5 ± 3.5
8	60	65.0 ± 2.8
10	20	45.5 ± 0.7
10	60	70.0 ± 0.0

A: ultrasonic amplitude; ΔT: temperature variation.

Table S2. Energy variation in the UAE from BRP.

A (%)	Energy (Wh)									
	Extraction time (min)									
	1	2	3	4	5	6	7	8	9	10
**20**	0.423	0.843								
**60**	1.425	2.741								
**100**	2.402	4.605								
**20**	0.416	0.832	1.254	1.658						
**60**	1.417	2.738	3.933	5.011						
**100**	2.378	4.576	6.531	8.210						
**20**	0.416	0.852	1.244	1.663	2.075	2.491				
**60**	1.433	2.739	3.891	4.941	5.895	6.814				
**100**	2.339	4.488	6.376	7.849	9.131	10.399				
**20**	0.412	0.830	1.246	1.656	2.065	2.473	2.881	3.290		
**60**	1.378	2.651	3.827	4.889	5.815	6.685	7.545	8.292		
**20**	0.420	0.836	1.248	1.658	2.068	2.477	2.836	3.304	3.693	4.097
**60**	1.429	2.717	3.935	5.042	6.030	6.862	7.790	8.632	9.474	10.267
